# A Robust INS/SRS/CNS Integrated Navigation System with the Chi-Square Test-Based Robust Kalman Filter

**DOI:** 10.3390/s20205909

**Published:** 2020-10-19

**Authors:** Guangle Gao, Shesheng Gao, Genyuan Hong, Xu Peng, Tian Yu

**Affiliations:** 1 School of Automation, Northwestern Polytechnical University, Xi’an 710072, China; gshshnpu@nwpu.edu.cn (S.G.); 807971326@mail.nwpu.edu.cn (G.H.); tianyu15804057252@mail.nwpu.edu.cn (T.Y.); 2 Research & Development Institute, Northwestern Polytechnical University, Shenzhen 518057, China

**Keywords:** robustness, noise estimation, chi-square test, integrated navigation, redshift navigation system

## Abstract

In order to achieve a highly autonomous and reliable navigation system for aerial vehicles that involves the spectral redshift navigation system (SRS), the inertial navigation (INS)/spectral redshift navigation (SRS)/celestial navigation (CNS) integrated system is designed and the spectral-redshift-based velocity measurement equation in the INS/SRS/CNS system is derived. Furthermore, a new chi-square test-based robust Kalman filter (CSTRKF) is also proposed in order to improve the robustness of the INS/SRS/CNS navigation system. In the CSTRKF, the chi-square test (CST) not only detects measurements with outliers and in non-Gaussian distributions, but also estimates the statistical characteristics of measurement noise. Finally, the results of our simulations indicate that the INS/SRS/CNS integrated navigation system with the CSTRKF possesses strong robustness and high reliability.

## 1. Introduction

For hypersonic cruise vehicles (HCVs), a highly autonomous and reliable navigation system is needed [1,2]. The inertial navigation system (INS) is one of the most widely used navigation systems [3,4,5]. The INS is a self-contained system and can provide highly accurate positions, velocities, and attitudes for short-term navigation. However, the gyro drift and accelerometer bias lead to unbounded error growth in the INS [5]. In order to overcome this shortcoming, the inertial navigation system/global navigation satellite system(INS/GNSS) integrated navigation system has been investigated [6,7,8]. However, the GNSS relies on signals from artificial satellites, and therefore lacks autonomy and is susceptible to artificial interference [9]. The celestial navigation system (CNS) is an autonomous navigation system that has lower positioning accuracy than the GNSS but has the advantage of not accumulating navigation error and a strong ability to resist electromagnetic interference [10,11,12]. Thus, researchers have also investigated the INS/CNS integrated navigation system, which incorporates the measurement information from the CNS to correct the deviations in the INS. However, the CNS also has its defects, including the difficulty of star selection and outdated data.

The spectral redshift navigation system (SRS) is a novel application in the navigation field. In the SRS, velocity can be obtained from the spectral redshift information of celestial spectra. Compared with other navigation systems, the SRS has the advantage of simple navigation principles, easy star selection, and no time delay [13]. In order to improve the autonomy of the integrated navigation of spacecraft, the SRS is widely used as an auxiliary navigation system to assist in correcting the velocity error of the main navigation system. For example, the authors of [13] used both the CNS and the SRS to correct the divergence caused by model errors of the orbital dynamics equations used in deep space exploration. The authors of [14] investigated the INS/SRS/GNS integrated navigation system, which used geomagnetic navigation and spectral redshift navigation to correct for the error of the INS. Thus, for HCVs, the SRS can also correct for the bias of the INS, avoiding parameter divergence while maintaining system autonomy.

To keep the reliability and improve the accuracy of the output of an integrated navigation system, information fusion is also important. The conventional Kalman filter (KF) has been a primary algorithm for linear navigation system integration [15,16]. However, in order to achieve information fusion when using the traditional KF, the accuracy system model and exact noise statistics are required [17]. In reality, the system always involves uncertainties caused by outliers in measurements under highly dynamic conditions. Thus, the authors of [18] proposed a sigma-point-based receding horizon Kalman filter (SPRHKF) to improve robustness. However, since this filter is based on a finite impulse response structure, the filtering convergence is poor [19]. The Sage–Husa noise statistic estimator has also been used to develop an adaptive KF [20,21]. However, the forgetting factors used in these filters are determined empirically. In [22,23], an H-infinity strategy was used to handle the uncertainties in observation noise. However, this method may only work under the condition of randomly occurring outliers. Additionally, the Huber-based KF has been applied to resist the influences of measurement outliers through the statistical linear regression of nonlinear system functions [24]. However, this method achieves its robustness by sacrificing accuracy. The authors of [25,26] also estimated scaling factors for the covariance of measurement noise to further adjust the Kalman gain to maintain robustness. However, this method may lead to a suboptimal filtering solution because the scaling factors are determined empirically. Furthermore, the hypothesis test method is great at detecting the changes in observations with outliers; examples of such tests include the chi-square test (CST) [27] and the generalized likelihood ratio test (GLRT) [28,29]. However, many studies simply investigated the hypothesis test method as a fault detection and isolation method to remove all observations with outliers but did not utilize the useful information in those observations, leading to the loss of navigation accuracy [30,31].

Thus, based on the above research, this paper deduces the linear relationship equation based on velocity in the east-north-up frame and the redshift of the observed vehicle, and then establishes the INS/SRS/CNS integrated navigation model. Meanwhile, to improve robustness, the chi-square test-based robust Kalman filter (CSTRKF) is proposed. In the CSTRKF, the CST is used to detect the change in noise based on the innovation sequence. Furthermore, based on the judgment index of the CST, a robust noise estimator is also proposed. Finally, the results of our simulations indicate the CSTRKF has great robustness performance and the enhanced INS/SRS/CNS integrated navigation system with the CSTRKF has great reliability.

## 2. Relationship between Velocity and Redshift in the East-North-Up Geographical Frame

According to the redshift principle of a spectrum and the Doppler frequency shift formula, we can obtain the following equation [13]:

(1) z=1+vr/c1−(vp−vc)2c2−1

where z denotes the spectral redshift value of the celestial body calculated in the target vehicle; $v_{p}$ denotes the velocity vector of the vehicle in the inertial frame (I-frame); $v_{c}$ denotes the velocity vector of the celestial body in the I-frame, which can be obtained by querying the celestial ephemeris; c is the velocity of light; and $v_{r}$ denotes the radial velocity along the direction from the target vehicle to the observed celestial body.

Assuming that the aircraft can obtain the spectral redshift values of three noncollinear observed celestial bodies, the three-vector operation relationship can be written as

(2) $$\left\{\begin{array}{l} v_{r1}=(v_{p}-v_{c1})\cdot u_{1}\\ v_{r2}=(v_{p}-v_{c2})\cdot u_{2}\\ v_{r3}=(v_{p}-v_{c3})\cdot u_{3} \end{array}\right.$$

where $\left(v_{c1},v_{c2},v_{c3}\right)$ represents the velocity vectors of three reference celestial bodies in the I-frame, which can be obtained by querying the ephemeris of related celestial bodies; and $\left(u_{1},u_{2},u_{3}\right)$ represents the unit vector of the position vector of each celestial body pointing to the aircraft in the inertial coordinate system, which can be measured by the star sensor.

Then, substituting (2) into (1) produces

(3) $$\left\{\begin{array}{l} (v_{p}-v_{1})\cdot u_{1}-(1+z_{1})\sqrt{c^{2}-{\left|v_{p}-v_{1}\right|}^{2}}+c=0\\ (v_{p}-v_{2})\cdot u_{2}-(1+z_{2})\sqrt{c^{2}-{\left|v_{p}-v_{2}\right|}^{2}}+c=0\\ (v_{p}-v_{3})\cdot u_{3}-(1+z_{3})\sqrt{c^{2}-{\left|v_{p}-v_{3}\right|}^{2}}+c=0 \end{array}\right.$$

Because (3) is a nonlinear equation, it should be linearized with the Taylor expansion in order to solve for $v_{p}$.

According to (3), set the function as

(4) $$Z_{i}(v_{p})=(v_{p}-v_{ci})\cdot u_{i}-(1+z_{i})\sqrt{c^{2}-{\left|v_{p}-v_{i}\right|}^{2}}+c$$

where *i* represents different observed objects.

Then, the first-order Taylor expansion of (4) yields

(5) $$Z_{i}(v_{p})=Z_{i}(v_{p}){|}_{v_{p}=0}+\frac{\partial Z_{i}(v_{p})}{\partial v_{px}}{|}_{v_{p}=0}\cdot v_{px}+\frac{\partial Z_{i}(v_{p})}{\partial v_{py}}{|}_{v_{p}=0}\cdot v_{py}+\frac{\partial Z_{i}(v_{p})}{\partial v_{pz}}{|}_{v_{p}=0}\cdot v_{pz}+\Delta_{Z}$$

where $\Delta_{Z}$ represents the higher-order term and ${\left(v_{px},v_{py},v_{pz}\right)}^{T}$ represents the components of $v_{p}$ in the I-frame.

After omitting the higher-order terms in Equation (5), the equation can be rewritten as

(6) $$\left\{ \begin{array}{l} Z_{1}(0)+\frac{\partial Z_{1}(v_{p})}{\partial v_{px}}{|}_{v_{p}=0}\cdot v_{px}+\frac{\partial Z_{1}(v_{p})}{\partial v_{py}}{|}_{v_{p}=0}\cdot v_{py}+\frac{\partial Z_{1}(v_{p})}{\partial v_{pz}}{|}_{v_{p}=0}\cdot v_{pz}=0\\ Z_{2}(0)+\frac{\partial Z_{2}(v_{p})}{\partial v_{px}}{|}_{v_{p}=0}\cdot v_{px}+\frac{\partial Z_{2}(v_{p})}{\partial v_{py}}{|}_{v_{p}=0}\cdot v_{py}+\frac{\partial Z_{3}(v_{p})}{\partial v_{pz}}{|}_{v_{p}=0}\cdot v_{pz}=0\\ Z_{3}(0)+\frac{\partial Z_{3}(v_{p})}{\partial v_{px}}{|}_{v_{p}=0}\cdot v_{px}+\frac{\partial Z_{3}(v_{p})}{\partial v_{py}}{|}_{v_{p}=0}\cdot v_{py}+\frac{\partial Z_{3}(v_{p})}{\partial v_{pz}}{|}_{v_{p}=0}\cdot v_{pz}=0 \end{array}\right.$$

Then, a nonhomogenous equation can be obtained and written as

(7) $$\left[\begin{array}{ccc} \frac{\partial Z_{1}}{\partial v_{px}}{|}_{v_{p}=0} & \frac{\partial Z_{1}}{\partial v_{py}}{|}_{v_{p}=0} & \frac{\partial Z_{1}}{\partial v_{pz}}{|}_{v_{p}=0}\\ \frac{\partial Z_{2}}{\partial v_{px}}{|}_{v_{p}=0} & \frac{\partial Z_{2}}{\partial v_{py}}{|}_{v_{p}=0} & \frac{\partial Z_{2}}{\partial v_{pz}}{|}_{v_{p}=0}\\ \frac{\partial Z_{3}}{\partial v_{px}}{|}_{v_{p}=0} & \frac{\partial Z_{3}}{\partial v_{py}}{|}_{v_{p}=0} & \frac{\partial Z_{3}}{\partial v_{pz}}{|}_{v_{p}=0} \end{array}\right]v_{p}^{}=Lv_{p}^{}=\left[\begin{array}{c} -Z_{1}(0)\\ -Z_{2}(0)\\ -Z_{3}(0) \end{array}\right].\;$$

Because the three observed celestial bodies are noncollinear, L is a full rank matrix. Thus, it has

(8) $$v_{p}^{}==-\left[\begin{array}{c} Z_{1}(0)\\ Z_{2}(0)\\ Z_{3}(0) \end{array}\right]L^{-1}.\;$$

Therefore, the velocity of aircraft calculated by the SRS in the ENU-frame can be obtained and written as

(9) $$v_{\mathrm{SRS}}=C_{g}^{i}v_{p}=-C_{e}^{i}C_{g}^{e}\left[\begin{array}{c} Z_{1}(0)\\ Z_{2}(0)\\ Z_{3}(0) \end{array}\right]L^{-1}$$

where, $v_{\mathrm{SRS}}$ is the velocity calculated by the SRS in the east-north-up geographical frame (ENU-frame); $C_{g}^{e}$ is the conversion matrix from the Earth-frame to the ENU-frame; and $C_{e}^{i}$ is the conversion matrix from the I-frame to the Earth-frame.

## 3. Model of the INS/SRS/CNS Integrated Navigation System

**Figure 1:**
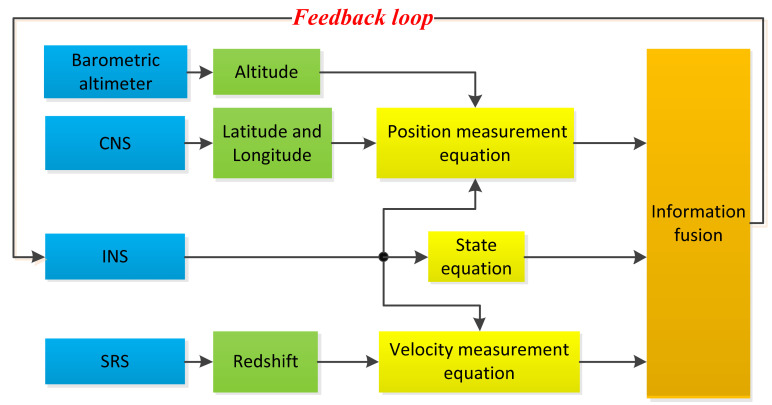
Structure of the inertial navigation system (INS)/spectral redshift navigation system (SRS)/celestial navigation system (CNS) integrated navigation system.

The structure of the INS/SRS/CNS integrated navigation system is shown in Figure 1. In the INS/SRS/CNS integrated navigation system, the INS is the main system, and the SRS, the CNS, and the barometric altimeter provide the velocity and position measurements to help correct the deviation of the INS. In addition, a closed loop system is set in the INS/SRS/CNS integrated system, which can further improve the system’s accuracy.

### 3.1. Kinematic Model of the INS/SRS/CNS Integrated Navigation System

According to the error model of the INS, we can represent the kinematic model of the integrated navigation system as [14]

(10) $$\overset{\cdot }{X}(t)=F(t)X(t)+W(t)$$

where X(t) is the system state vector, specifically represented as

(11) $$X(t)=[\phi_{E}\;\;\phi_{N}\;\;\phi_{U}\;\;\delta v_{E}\;\;\delta v_{N}\;\;\delta v_{U}\;\;\delta L\;\;\delta \lambda \;\;\delta h\;\;\;\;\varepsilon_{E}^{b}\;\;\varepsilon_{N}^{b}\;\;\varepsilon_{U}^{b}\;\;\nabla_{E}\;\;\nabla_{N}\;\;\nabla_{U}{]}^{T}.$$

$\left(\phi_{E},\phi_{N},\phi_{U}\right)$ denotes the platform angle error in the ENU-frame; $\left(\delta v_{E},\delta v_{N},\delta v_{U}\right)$ denotes the velocity error in the ENU-frame; (δL,δλ,δh) denotes the position error in the ENU-frame; and $\left(\varepsilon_{E}^{b},\varepsilon_{N}^{b},\varepsilon_{U}^{b}\right)$ and $\left(\nabla_{E},\nabla_{N},\nabla_{U}\right)$ respectively denote the gyro random drift and the accelerometer random bias.

F(t) is the system matrix, which is specifically represented as [17]:

(12) $$F(t)={\left[\begin{array}{cc} F_{N} & F_{S}\\ 0_{6\times 9} & 0_{6\times 6} \end{array}\right]}_{15\times 15}$$

where $F_{N}$ is the attitude, velocity, and position-related system submatrix and $F_{S}$ is the gyro and accelerometer-related system submatrix.

W(t) is the system noise matrix, specifically

(13) $$W(t)={\left[\begin{array}{cccccc} w_{E}^{g} & w_{N}^{g} & w_{U}^{g} & w_{E}^{a} & w_{N}^{a} & w_{U}^{a} \end{array}\right]}^{T}$$

where $\left(w_{E}^{g},w_{N}^{g},w_{U}^{g}\right)$ indicates the random error vector of gyroscopes and $\left(w_{E}^{a},w_{N}^{a},w_{U}^{a}\right)$ indicates the accelerometer drift vector.

### 3.2. Measurement Model of the INS/SRS/CNS Integrated Navigation System

The velocity measurement equation based on the SRS can be expressed as

(14) $$Z_{k,}{}_{\mathrm{SRS}}=v_{\mathrm{INS}}-v_{\mathrm{SRS}}=\left[\begin{array}{l} \delta v_{E}\\ \delta v_{N}\\ \delta v_{H} \end{array}\right]+V_{k,\mathrm{SRS}}$$

where $v_{\mathrm{INS}}$ is the velocity obtained by the INS in the ENU-frame and $V_{SRS}$ is the noise matrix of the SRS.

The longitude and latitude observation equation of the INS/SRS/CNS is the difference of the longitude and latitude information between the INS and the CNS, which is shown as

(15) $$Z_{k,\mathrm{CNS}}=\left[\begin{array}{l} \lambda_{\mathrm{INS}}-\lambda_{\mathrm{CNS}}\\ L_{\mathrm{INS}}-L_{\mathrm{CNS}} \end{array}\right]=\left[\begin{array}{l} \delta \lambda \\ \delta L \end{array}\right]+V_{k,\mathrm{CNS}}$$

where $\left(\lambda_{\mathrm{CNS}},L_{\mathrm{CNS}}\right)$ denotes the longitude and latitude measurement of the CNS in the ENU-frame; $\left(\lambda_{\mathrm{INS}},L_{\mathrm{INS}}\right)$ denotes the longitude and latitude outputs of the INS in the ENU-frame; and $V_{\mathrm{CNS}}$ denotes the measurement noise matrix of the CNS.

In order to prevent the divergence of the altitude channel of the INS, the barometric altimeter is introduced into the integrated navigation system. Then, the measurement equation of altitude is shown as

(16) $$Z_{h}=\left[h_{\mathrm{INS}}-h_{\mathrm{BA}}\right]=\delta h+V_{\mathrm{BA}}$$

where $h_{\mathrm{INS}}$ and $h_{\mathrm{BA}}$ denote the altitude output by the INS and the barometric altimeter in the ENU-frame, respectively, and $V_{\mathrm{BA}}$ denotes the measurement noise matrix of the barometric altimeter.

Finally, the whole measurement equation of the INS/SRS/CNS system can be written as

(17) $$Z_{k}=H_{k}X_{k}+V_{k}$$

where $H_{k}=\left[\begin{array}{ccc} 0_{6\times 3} & I_{6\times 6} & 0_{6\times 6} \end{array}\right]$ is the measurement matrix of the INS/SRS/CNS system; $X_{k}$ is the discrete state vector; and $V_{k}\;=\;[V_{k,\mathrm{SRS}};V_{k,\mathrm{CNS}};V_{k,\mathrm{BA}}]$ is the measurement noise matrix of the whole system.

## 4. The Chi-Square Test-Based Robust Kalman Filter

### 4.1. The Traditional Kalman Filter

The denotation of the noise matrices is as follows:

(18) $$\left\{\begin{array}{l} E[W_{k}]=0,E[W_{k}W_{j}]=Q_{k}\delta_{kj}\\ E[V_{k}]=0,E[V_{k}V_{j}]=R_{k}\delta_{kj}\\ E[W_{k}V_{k}]=0 \end{array}\right.$$

where $Q_{k}$ is the non-negative matrix, $R_{k}$ is the positive matrix, and $\delta_{kj}$ is the Kronecker-δ function.

Then, the procedure of the KF can be written as follows:

First, the state prediction is shown as

(19) $$X_{k|k-1}=F{\hat{X}}_{k-1}+W_{k-1}$$

(20) $$P_{k|k-1}=FP_{k-1}F^{T}+Q$$

(21) $$Z_{k|k-1}=H_{k}{\hat{X}}_{k|k-1}$$

(22) $$P_{k|k-1}^{zz}=H_{k}P_{k|k-1}H_{k}^{T}+R_{k}$$

where $X_{k|k-1}\in R^{n}$ denotes the state prediction; $P_{k|k-1}\in R^{n\times n}$ denotes the state prediction covariance matrix; $Z_{k|k-1}\in R^{m}$ denotes the measurement prediction; and $P_{k|k-1}^{zz}\in R^{m\times m}$ denotes the predicted measurement covariance matrix.

Second, the state estimation is shown as

(23) $$K_{k}=P_{k/k-1}H_{k}^{T}P_{k|k-1}^{zz}{}^{-1}$$

(24) $${\hat{X}}_{k}=X_{k/k-1}+K_{k}(Z_{k}-Z_{k|k-1}-r_{k})$$

(25) $$P_{k}=(I-K_{k}H_{k})P_{k/k-1}$$

where ${\hat{X}}_{k}$ denotes the state estimation and $P_{k}$ denotes the estimation covariance matrix of the state.

### 4.2. CST-Based Noise Estimator for Measurement

In reality, the measurement noise is unknown and changes with time; thus, it needs to be estimated and adjusted to maintain the robustness and accuracy of the estimation obtained from the Kalman filter. In this paper, a new noise estimator based on the CST is proposed:

Assuming $\left\{\nu_{j}|j=k-M+1,\cdots ,k\right\}$ is the selected independent innovation sequence at time k under a limited window of size *M*, the innovation-based measurement is calculated as

(26) $$\nu_{k}=Z_{k}-H_{k}X_{k/k-1}.\;$$

The hypothesis test based on the innovation sequence can be set as

(27) $$\left\{\begin{array}{l} H_{0}:\;E[\nu \nu^{T}]=P_{k|k-1}^{zz},\mathrm{charactoristic}\;\mathrm{of}\;\mathrm{noise}\;\mathrm{is}\;\mathrm{unchanged}\;\\ H_{1}:\;E[\nu \nu^{T}]=\Sigma_{k}\neq P_{k|k-1}^{zz},\;\mathrm{charactoristic}\;\mathrm{of}\;\mathrm{noise}\;\mathrm{is}\;\mathrm{changed} \end{array}\right.$$

where $P_{k|k-1}^{zz}$ represents the covariance of the prior innovation estimation by the Kalman filter and $\Sigma_{k}$ denotes the covariance of the posterior innovation estimation, which can be calculated under the limited innovation sequence as

(28) $$\sum_{k}^{}=\frac{1}{M}\sum_{j=1}^{M}\left(\nu_{k-j+1}-{\hat{\mu }}_{k}\right){\left(\nu_{k-j+1}-{\hat{\mu }}_{k}\right)}^{T}$$

where

(29) $${\hat{\mu }}_{k}={\hat{r}}_{k}=\frac{1}{M}\sum_{j=1}^{M}\nu_{k-j+1}.\;$$

As (27) shows, under the accurate system model, if a measurement is without an outlier, $\Sigma_{k}$ is near the value of $P_{k|k-1}^{zz}$. Otherwise, the statistical noise can be considered to have changed. Then, according to the principle of the CST, the judgment index can be expressed as

(30) $$\lambda (k)=\frac{1}{M}\sum_{j=1}^{M}{\left(\nu_{k-j+1}-{\hat{\mu }}_{k}\right)}^{T}{\left(P_{k|k-1}^{zz}\right)}^{-1}(\nu_{k-j+1}-{\hat{\mu }}_{k})$$

where $\lambda (k)∼\chi {\left(m\right)}^{2}$.

According to the hypothesis test, setting the significance level to α (0 < α < 1) with a threshold of *T* makes α follow [28]

(31) $$P\left\{\lambda (k)>T\right\}=\alpha .\;$$

When the statistical characteristics of measurement noise are unchanged compared to the last time, λ(k) will be small and under threshold *T*. Otherwise, the judgment index will be over the threshold, and in that time the covariance of measurement noise should be adjusted

Assuming

(32) $${\hat{R}}_{k}=\beta_{k}{\hat{R}}_{k-1}$$

where $\beta_{k}$ is the adjust matrix of the measurement noise matrix.

Additionally, substituting (32) with (30), one obtains

(33) $$\lambda (\beta_{k})=\frac{1}{M}\sum_{j=1}^{M}{\left(\nu_{k-j+1}-{\hat{\mu }}_{k}\right)}^{T}{\left({\left(H_{k}P_{k|k-1}H_{k}^{T}+\beta_{k}{\hat{R}}_{k-1}\right)}^{-1}\right)}^{-1}(\nu_{k-j+1}-{\hat{\mu }}_{k}).$$

Then, set the equation as follows

(34) $$N(\beta_{k})=\lambda (\beta_{k})-T.$$

According to Newton’s method, one then obtains

(35) $$\beta_{k}(i+1)=\beta_{k}(i)+N(\beta_{k}(i))/\frac{\partial N(\beta_{k}(i))}{\partial \beta_{k}(i)}.$$

Thus, we can obtain

(36) $$\beta_{k}(i+1)=\beta_{k}(i)+\frac{\frac{1}{M}\sum_{j=1}^{M}{\left(\nu_{k-j+1}-{\hat{\mu }}_{k}\right)}^{T}{\left({\left(H_{k}P_{k|k-1}H_{k}^{T}+\beta_{k}(i){\hat{R}}_{k-1}\right)}^{-1}\right)}^{-1}(\nu_{k-j+1}-{\hat{\mu }}_{k})-T}{\frac{1}{M}\sum_{j=1}^{M}{\left(\nu_{k}-{\hat{\mu }}_{k}\right)}^{T}{\left(H_{k}P_{k|k-1}H_{k}^{T}+\beta_{k}(i){\hat{R}}_{k-1}\right)}^{-1}{\hat{R}}_{k-1}{\left(H_{k}P_{k|k-1}H_{k}^{T}+\beta_{k}(i){\hat{R}}_{k-1}\right)}^{-1}(\nu_{k}-{\hat{\mu }}_{k})}$$

where *i* denotes the time of iterations in Newton’s method.

Finally, set the initial $\beta_{k}(1)=1$ and keep the iterations of (36) until $\lambda (\beta_{k}(i))$ is under threshold *T*.

Accordingly, $\beta_{k}$ can be written as

(37) $$\beta_{k}=\left\{\begin{array}{l} \beta_{k}(i),\;\lambda (\beta_{k}(i))<T\\ continuetheiteration,\;\mathrm{Others} \end{array}\right..\;$$

**Remark 1**: *Further, to avoid an unlimited number of iterations, a cut off time of C = 20 is set in the CSTRKF. If the $\lambda (\beta_{k}(i))$ is not under the threshold when the number of iterations is over C, the iterations end and the measurement update in the CSTRKF stops at this time.*

In order to avoid the element of ${\hat{R}}_{k}$ being negative and keep ${\hat{R}}_{k}$ as a diagonal matrix, $\beta_{k}^{}$ is modified as

(38) $$\beta_{k}^{*}=\mathrm{diag}(\beta_{1,k}^{*},\beta_{2,k}^{*},\cdots ,\beta_{m,k}^{*})$$

where

(39) $$\beta_{i,k}^{*}=\max\{\varepsilon ,\beta_{k}(i,i)\},i=1,2,\cdots ,m$$

where ε is smaller than 1.

Therefore, the covariance estimation of the observation noise can be written as

(40) $${\hat{R}}_{k}=\beta_{k}^{*}{\hat{R}}_{k-1}.\;$$

### 4.3. Procedure of the CSTRKF

**Figure 2:**
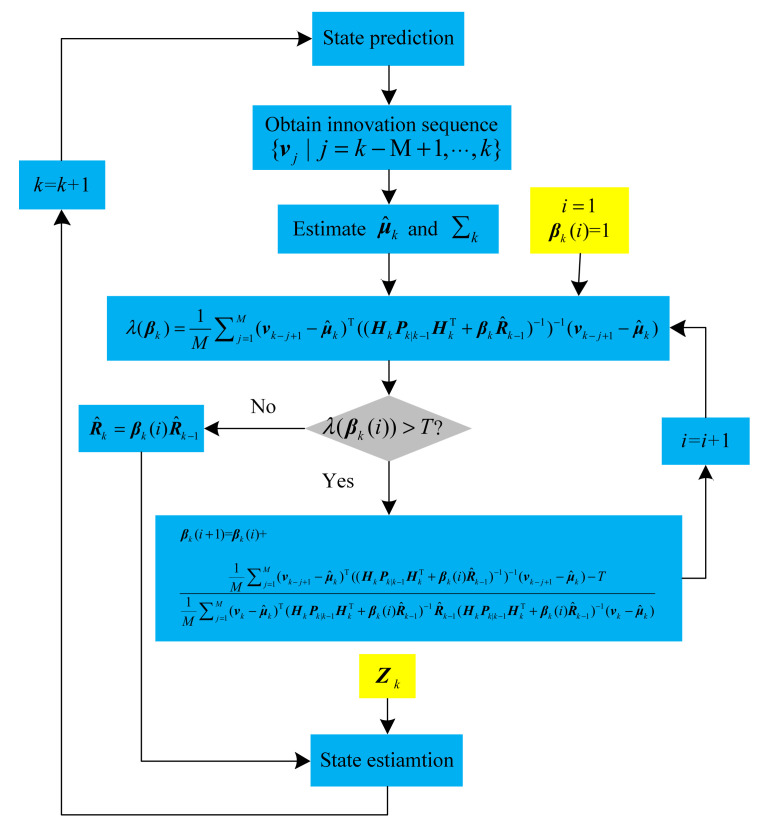
Procedure of the Chi-Square Test-Based Robust Kalman Filter.

By involving the CST-based noise estimator in the KF, the CSTRKF can be obtained, as illustrated in Figure 2.

As Figure 2 shows, the procedure is as follows:

Step 1. Initialize the matrix of $X_{0}$, $P_{0}$, $R_{0}$, and $Q_{0}^{}$;

Step 2. Achieve the prediction of $X_{k|k-1}$, $P_{k|k-1}$, $Z_{k|k-1}$, and $P_{k|k-1}^{zz}$ by using (19) through (22);

Step 3. Compute the innovations sequence using (26);

Step 4. Set $\beta_{k}(1)=1$ and calculate the judgment index from (30);

Step 5. Then judge whether ${\hat{R}}_{k}$ is changed by CST: if  λ(k)≤T, ${\hat{R}}_{k-1}$ is considered as accurate and $\beta_{k}^{}$ is adjusted to 1. Otherwise, ${\hat{R}}_{k-1}$ is considered as changed and $\beta_{k}^{}$ needs to be iterated by (36) until $\lambda (\beta_{k}(i))$ is under threshold T;

Step 6. Calculate ${\hat{R}}_{k}$ using (40);

Step 7. Incorporate the new ${\hat{R}}_{k}$ and estimate ${\hat{X}}_{k}$ and ${\hat{P}}_{k}$ using (23)–(25); and

Step 8. Repeat steps 2–7 until the navigation ends.

## 5. Simulation and Results

**Figure 3:**
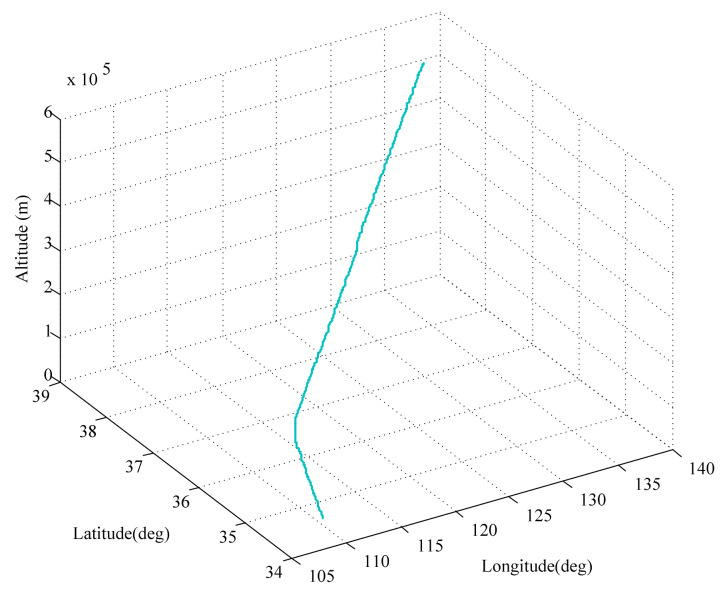
Dynamic flight trajectory of hypersonic cruise vehicles (HCVs).

In this section, the superiority of the INS/SRS/CNS integrated system with the proposed CSTRKF algorithm is verified through simulations. Figure 3 shows the dynamic flight trajectory of HCVs. The parameters of the simulations are shown in Table 1. The total simulation time was set to 30 min (1800 s) and the filtering period was 0.1 s. In the CSTRKF, the significance level α was 0.05.

### 5.1. Evaluation of CSTRKF under the Condition of Measurements with Outliers

In this part, the CSTRKF is compared with the H-infinity-based robust filter (HI-RF) [22] and the traditional KF under the condition of measurements with outliers in the INS/SRS/CNS integrated navigation system.

**Figure 4:**
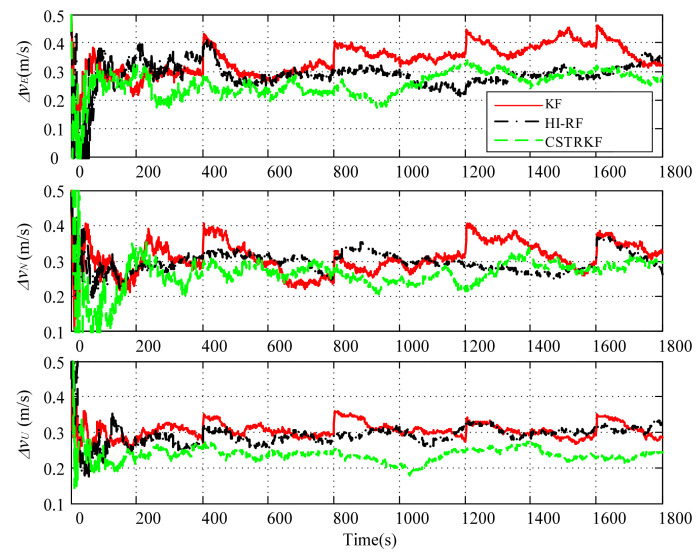
Velocity error of the INS/SRS/CNS integrated system with different filters under the condition of measurements with outliers.

**Figure 5:**
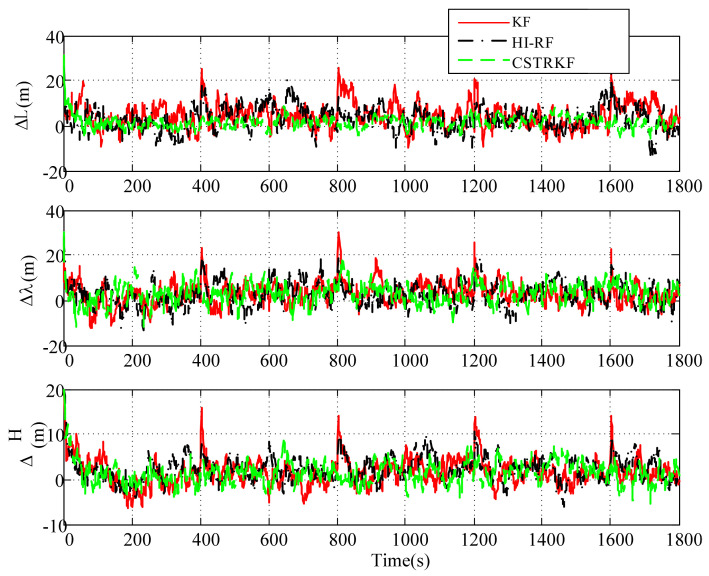
Position error of the INS/SRS/CNS integrated system with different filters under the condition of measurements with outliers.

The observation errors were enlarged to 4 times their normal error for observations at 400s, 800s, 1200s, and 1600s. Under those observations with outliers, the curves of the velocity error and position error under the traditional KF, the HI-RF, and the CSTRKF are compared in Figure 4 and Figure 5.

From Figure 4 and Figure 5, it can be seen that under the KF, the velocity errors and position errors have the largest fluctuations and values at times 400s, 800s, 1200s, and 1600s when compared with those under the other two filters, indicating the poor robustness of the KF, which does not involve the measurement noise estimation method. Additionally, due to the utilization of the H-infinity strategy to resist outliers, the velocity errors and position errors under the HI-RF are smaller than those under the KF. However, this method still has pronounced errors. Furthermore, it can be seen in Figure 4 and Figure 5 that by using the CST to judge the change in measurement noise and estimate the noise simultaneously, the velocity and position errors have the smallest values among the three filters, which shows the great robust performance of the CSTRKF.

The root-mean-square error (RMSE) and mean absolute error (MAE) are defined as

(41) $$\mathrm{RMSE}(\Delta x)=\sqrt{\frac{1}{T}\sum_{k=1}^{T}{\left[\Delta x(k)\right]}^{2}}$$

(42) $$\mathrm{MAE}(\Delta x)=\frac{1}{T}\sum_{k=1}^{T}\Delta x(k)$$

where *k* denotes the simulation times and Δx denotes the ΔV or ΔP, which is calculated as

(43) $$\Delta V=\sqrt{\Delta v_{E}^{2}+\Delta v_{N}^{2}+\Delta v_{U}^{2}}$$

(44) $$\Delta P=\sqrt{\Delta L_{}^{2}+\Delta \lambda_{}^{2}+\Delta H_{}^{2}}$$

The MAEs of velocity and position at times with outliers and at times without outliers are shown in Table 2. When measurements have outliers, under the KF, the system has the greatest MAEs for both velocity and position, approximately 0.5443 m/s and 25.0624 m, respectively. By utilizing the H-infinity strategy, the MAEs of both velocity and position under the HI-RF are smaller than those under the KF by 13.1% and 22%, respectively. Furthermore, thanks to the estimation noise based on the CST, the MAEs of velocity and position under the CSTRKF are smaller than those under the HI-RF by 10.2% and 28.2%, respectively, which shows the superiority of the CSTRKF.

**Figure 6:**
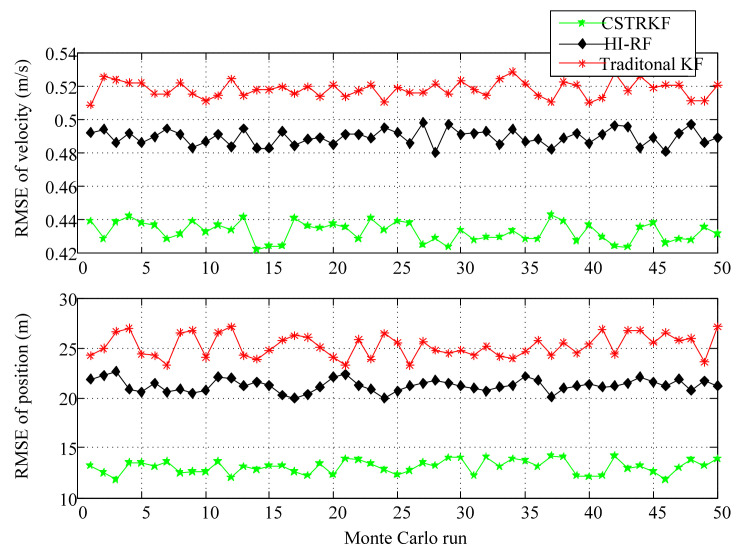
Root-mean-square errors (RMSEs) of velocity and position in the INS/SRS/CNS integrated system with different filters at the times when measurements have outliers.

**Figure 7:**
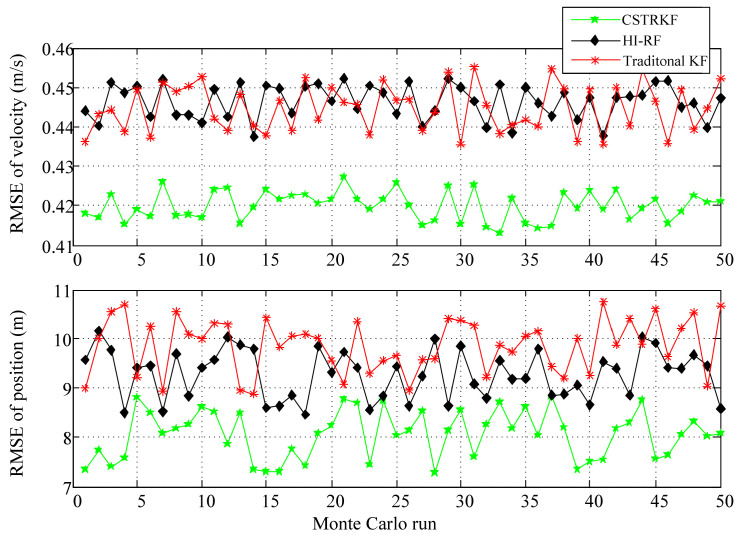
RMSEs of velocity and position in the INS/SRS/CNS integrated system with different filters at the times when measurements do not have outliers.

To further evaluate the performance of the CSTRKF, a Monte Carlo simulation was run 50 times. The RMSEs of the velocity and position errors of the INS/SRS/CNS integrated system under different filters are shown in Figure 6 and Figure 7. As Figure 6 and Figure 7 show, when measurements have outliers, the RMSEs of the velocity and position under the CSTRKF are in the ranges of 0.42–0.44 m/s and 11.88–14.19 m, respectively, and are smaller than those of the velocity and position errors in the HI-RF (0.48–0.49 m/s and 19.99–21.26 m, respectively) and in the KF (0.51–0.53 m/s and 23.24–27.16 m, respectively). Furthermore, due to the pronounced errors delivered through the filters, when measurements do not have outliers, the RMSEs of the velocity and position errors under the CSTRKF are in the ranges of 0.41–0.43 m/s and 7.28–8.84 m, which are also smaller than those of the velocity and position errors in the HI-RF (0.435–0.455 m/s and 8.46–10.13 m, respectively) and in the KF (0.438–0.452 m/s and 8.88–10.75 m, respectively).

### 5.2. Evaluation of CSTRKF under a Contaminated Gaussian Measurement Noise Condition

To continue to evaluate the performance of the proposed CSTRKF in terms of the non-Gaussian characteristics of noise statistics, the measurement noise was set to change as a contaminated Gaussian distribution, which is as follows

(45) $$v_{k}∼\left(1-\eta \right)N\left(0,R_{k}\;\right)\;+\;\eta N\left(5,5R_{k}\;\right)$$

where η is set to follow a uniform distribution between 0 and 1.

**Figure 8:**
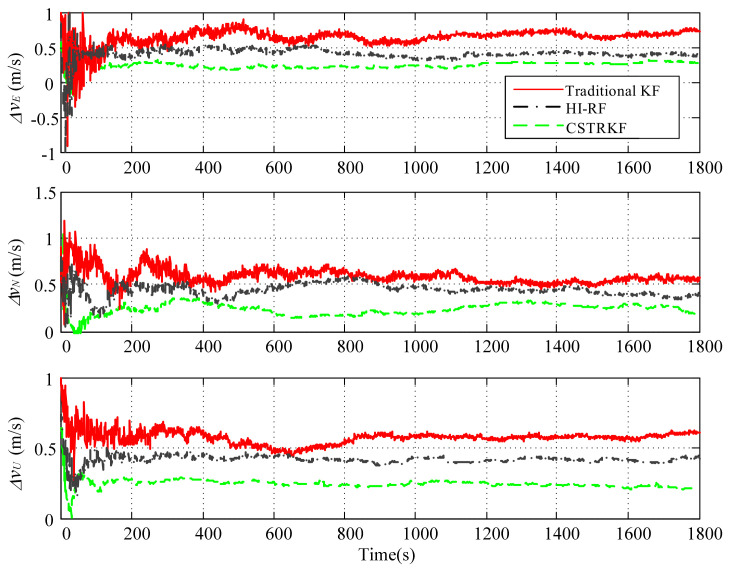
Velocity error of the INS/SRS/CNS integrated system with different filters under a contaminated Gaussian measurement noise condition.

**Figure 9:**
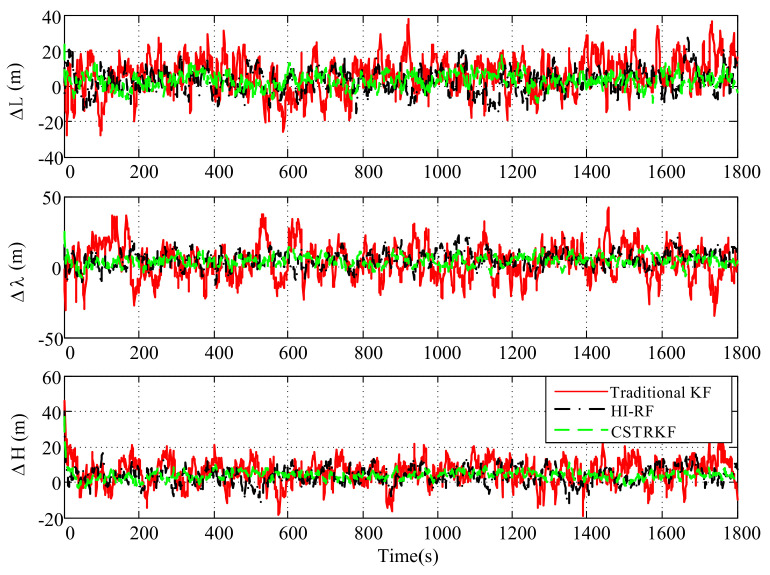
Position error of the INS/SRS/CNS integrated system with different filters under a contaminated Gaussian measurement noise condition.

Figure 8 and Figure 9 show the velocity error and position error under the KF, HI-RF, and CSTRKF. The MAE of velocity and position is shown in Table 3. From Figure 8 and Figure 9, it can be seen that without the relative method to deal with the contaminated Gaussian noise, the velocity error and position error under the KF reach their highest values across the entire set of simulations. Under the HI-RF, the system has a smaller velocity error and position error. Additionally, the MAE of velocity and position under the HI-RF are 37.5% and 27.2% smaller, respectively, than those under the KF. However, the velocity error and position error under the CSTRKF are the smallest, with MAEs 29.9% and 25.6% smaller, respectively, than those under the HI-RF, showing the superior performance of the CSTRKF under the condition of contaminated Gaussian noise.

**Figure 10:**
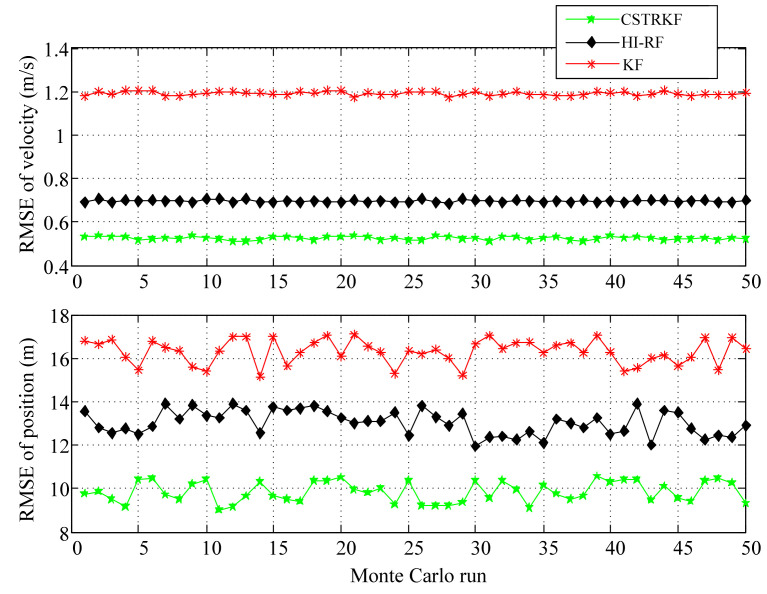
RMSEs of velocity and position measurements in the INS/SRS/CNS integrated system with different filters under a contaminated Gaussian measurement noise condition.

After 50 Monte Carlo simulations, the RMSEs of velocity error and position error of the INS/SRS/CNS integrated system under a contaminated Gaussian measurement noise condition were also calculated, as shown in Figure 10. As Figure 10 shows, under a contaminated Gaussian measurement noise condition, the RMSEs of the velocity and position errors under the CSTRKF are in the ranges of 0.51–0.54 m/s and 9.03–10.55 m, respectively, which is smaller than those of the velocity and position errors under the HI-RF (0.68–0.71 m/s and 11.99–13.91 m, respectively) and under the KF (1.18–1.22 m/s and 15.15–17.06 m, respectively), which also illustrates the superiority of the CSTRKF for information fusion.

## 6. Conclusions

In this paper, the linear relationship equation between the velocity in the ENU-frame and the redshift of an observed vehicle was deduced and the INS/SRS/CNS integrated navigation model was established based on this relationship for the purposes of improving the autonomy and reliability in the navigation of HCVs. Furthermore, to improve robustness, the CSTRKF algorithm was also proposed in the INS/SRS/CNS integrated navigation model. In the CSTRKF, based on the posterior innovation covariance estimation the CST has been added to the KF in order to detect the change in noise and estimate the statistical characteristics of measurement noise. The simulation results indicate that the CSTRKF has great robustness performance and, under the CSTRKF, the robustness of the INS/SRS/CNS integrated navigation system also improved.

## Figures and Tables

**Table 1:** Parameters of the Simulations.

Initial position	East longitude	108.9°
	North latitude	34.025°
	Altitude	60 km
Initial velocity	East	251 m/s
	North	251 m/s
	Up	225 m/s
Initial position error	East longitude	50 m
	North latitude	50 m
	Altitude	25 m
Initial velocity error	East	1 m/s
	North	1 m/s
	Up	1 m/s
Gyro parameters	Constant drift	0.5°/h
	White noise	0.5°/h
	Sampling frequency	10 Hz
Accelerometer parameters	Zero bias	0.1 mg
	White noise	0.1 mg
	Sampling frequency	10 Hz
SRS	Redshift measurement error	10^−8^
	Sampling frequency	1 Hz
CNS	Position measurement error	20 m
	Sampling frequency	1 Hz
Barometric altimeter	Altitude measurement error	10 m
	Sampling frequency	1 Hz

**Table 2:** Mean absolute error (MAE) of estimation under the condition of measurements with outliers.

Estimation	Filters	MAE	
		Times with Outlier	Times in Normal
Velocity	KF	0.5543 (m/s)	0.4509 (m/s)
	HI-KF	0.4817 (m/s)	0.4349 (m/s)
	CSTKF	0.4327 (m/s)	0.4236 (m/s)
Position	KF	25.0624 (m)	8.6598 (m)
	HI-KF	19.6607 (m)	8.3507 (m)
	CSTKF	14.1423 (m)	7.8930 (m)

**Table 3:** MAEs of estimation under the contaminated Gaussian noise condition.

Estimation	Filters	MAE
Velocity	KF	16.7923 (m)
	HI-KF	12.2325 (m)
	CSTKF	9.1047 (m)
Position	KF	1.1780 (m/s)
	HI-KF	0.7367 (m/s)
	CSTKF	0.5165 (m/s)
